# Malaria vector species composition and entomological indices following indoor residual spraying in regions bordering Lake Victoria, Tanzania

**DOI:** 10.1186/s12936-020-03452-w

**Published:** 2020-10-28

**Authors:** Charles Kakilla, Alphaxard Manjurano, Karen Nelwin, Jackline Martin, Fabian Mashauri, Safari M. Kinung’hi, Eric Lyimo, Doris Mangalu, Lucy Bernard, Nduka Iwuchukwu, Dismasi Mwalimu, Naomi Serbantez, George Greer, Kristen George, Richard M. Oxborough, Stephen M. Magesa

**Affiliations:** 1 National Institute for Medical Research, Mwanza, Tanzania; 2 PMI-AIRS Tanzania Project, Abt Associates, Mwanza, Tanzania; 3 National Malaria Control Program, Ministry of Health, Community Development, Gender, Elderly and Children, Dodoma, Tanzania; 4 U.S. President’s Malaria Initiative, Dar es Salaam, Tanzania; 5 U.S. President’s Malaria Initiative, U.S. Agency for International Development, Washington, DC USA; 6 PMI AIRS/VectorLink Project, Abt Associates, 6130 Executive Blvd, Rockville, MD 20852 USA

**Keywords:** Malaria vectors, Indoor residual spraying, Pirimiphos-methyl, Species composition, *Anopheles gambiae*, *Anopheles funestus*, *Anopheles arabiensis*, Seasonality, Tanzania

## Abstract

**Background:**

Vector control through long-lasting insecticidal nets (LLINs) and focal indoor residual spraying (IRS) is a major component of the Tanzania national malaria control strategy. In mainland Tanzania, IRS has been conducted annually around Lake Victoria basin since 2007. Due to pyrethroid resistance in malaria vectors, use of pyrethroids for IRS was phased out and from 2014 to 2017 pirimiphos-methyl (Actellic® 300CS) was sprayed in regions of Kagera, Geita, Mwanza, and Mara. Entomological surveillance was conducted in 10 sprayed and 4 unsprayed sites to determine the impact of IRS on entomological indices related to malaria transmission risk.

**Methods:**

WHO cone bioassays were conducted monthly on interior house walls to determine residual efficacy of pirimiphos-methyl CS. Indoor CDC light traps with or without bottle rotator were hung next to protected sleepers indoors and also set outdoors (unbaited) as a proxy measure for indoor and outdoor biting rate and time of biting. Prokopack aspirators were used indoors to capture resting malaria vectors. A sub-sample of *Anopheles* was tested by PCR to determine species identity and ELISA for sporozoite rate.

**Results:**

Annual IRS with Actellic® 300CS from 2015 to 2017 was effective on sprayed walls for a mean of 7 months in cone bioassay. PCR of 2016 and 2017 samples showed vector populations were predominantly *Anopheles arabiensis* (58.1%, n = 4,403 IRS sites, 58%, n = 2,441 unsprayed sites). There was a greater proportion of *Anopheles funestus *sensu stricto in unsprayed sites (20.4%, n = 858) than in sprayed sites (7.9%, n = 595) and fewer *Anopheles parensis* (2%, n = 85 unsprayed, 7.8%, n = 591 sprayed). Biting peaks of *Anopheles gambiae *sensu lato (*s.l*.) followed periods of rainfall occurring between October and April, but were generally lower in sprayed sites than unsprayed. In most sprayed sites, *An. gambiae s.l.* indoor densities increased between January and February, i.e., 10–12 months after IRS. The predominant species *An. arabiensis* had a sporozoite rate in 2017 of 2.0% (95% CI 1.4–2.9) in unsprayed sites compared to 0.8% (95% CI 0.5–1.3) in sprayed sites (p = 0.003). Sporozoite rates were also lower for *An. funestus* collected in sprayed sites.

**Conclusion:**

This study contributes to the understanding of malaria vector species composition, behaviour and transmission risk following IRS around Lake Victoria and can be used to guide malaria vector control strategies in Tanzania.

## Background

In sub-Saharan Africa, recent gains in malaria control have been mostly accomplished through a substantial boost in vector control using long-lasting insecticidal nets (LLINs) and indoor residual spraying (IRS). These tools have significantly contributed to a 50% reduction of *Plasmodium falciparum* infection prevalence in endemic countries between 2000 and 2015 [1]. IRS has been reported to successfully reduce malaria prevalence and incidence in several African countries in the past decade [2–4]. In mainland Tanzania, IRS implementation funded by the US President’s Malaria Initiative (PMI) was launched in 2007 in Muleba and Karagwe districts, located in Kagera Region. The initial locations were supported in response to a malaria epidemic in 2006 [5]. Thereafter, IRS activities with pyrethroid insecticides were progressively expanded to other districts in the Lake Victoria basin, including the remaining five districts of Kagera Region in 2009 and, in 2010 and 2011 to all 18 districts of Kagera, Mwanza, and Mara, covering 1.1 million structures and targeting nearly 6.3 million people [6].

Mosquito larvae collected around sites in the Lake Victoria basin in 2015 indicated that the malaria vector species composition varied by district with the predominant species being *Anopheles arabiensis* in Mara Region, Muleba and Ngara districts and *Anopheles gambiae *sensu stricto (*s.s*.) in Magu and Geita districts [7]. Pyrethroid resistance was documented by Kisinza et al. [7] in all districts that were tested in 2015 near Lake Victoria, including Musoma Rural, Magu, and Muleba. Due to the detection of pyrethroid resistance in malaria vectors, the use of pyrethroids for IRS was gradually phased out in accordance with WHO guidance that pyrethroids should be preserved for LLINs [8]. The carbamate insecticide, bendiocarb (Ficam®, 80% WP) was used alongside the pyrethroid deltamethrin K-Othrine® (WG 250) from 2011 to 2013 [5]. From 2014 to 2017, a long-acting organophosphate formulation of pirimiphos-methyl (Actellic® 300CS) was sprayed annually in all targeted areas of the Lake Victoria basin in the regions of Kagera, Geita, Mwanza and Mara.

Despite widespread pyrethroid resistance being detected in malaria vectors throughout Tanzania [9, 10], IRS in combination with pyrethroid LLINs have proven effective in mainland Tanzania [5, 11] and in Zanzibar [12]. Partly due to vector control, reported malaria deaths in mainland Tanzania reduced by ~ 32%, from 15,819 in 2010 to 5045 in 2016 [13].

Reported here are results of entomological surveillance covering 10 sprayed sites and 4 unsprayed control sites in the Lake Victoria Basin. The main objective was to evaluate the entomological impact of IRS with pirimiphos-methyl CS against malaria vectors. Specifically, entomological data were collected to assess the persistence of pirimiphos-methyl CS on sprayed walls, determine vector species composition, seasonality, feeding behaviour and *P. falciparum* infectivity.

## Methods

### Study area and duration

**Fig. 1:**
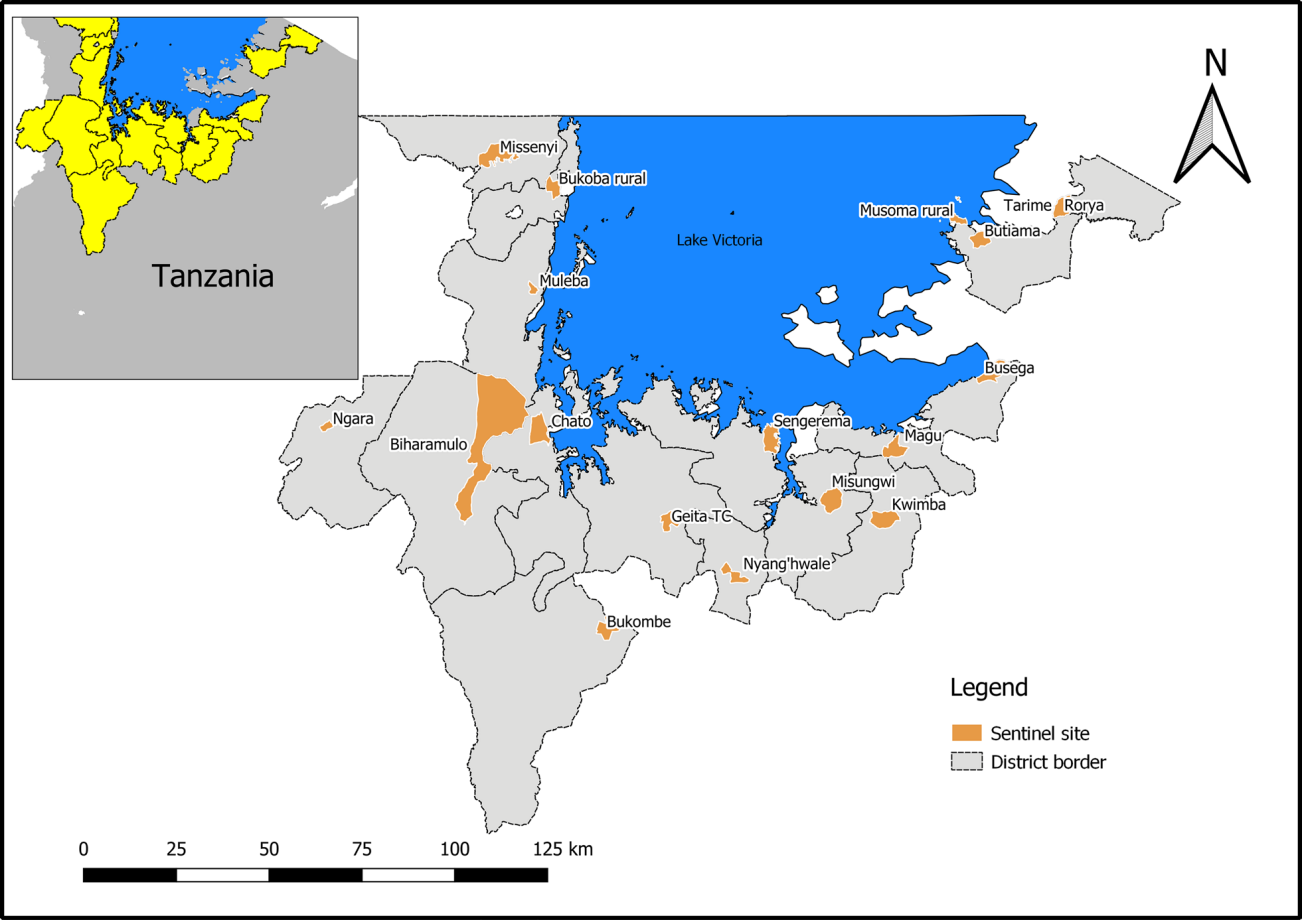
Study sites. Map of entomological surveillance sites in districts surrounding Lake Victoria, NW Tanzania. Showing all sites from entomological monitoring surveys conducted between 2015 and 2017

Entomological surveillance was conducted in regions around Lake Victoria, northwestern Tanzania. For 3 years from 2015 to 2017, between 8 and 10 districts of the Lake Victoria basin were sprayed annually with pirimiphos-methyl CS. Entomological monitoring of the insecticide decay rate, malaria vector densities, *Anopheles* species composition, and *P. falciparum* infectivity rates was conducted in sprayed and unsprayed sites. A list of districts and annual spray status is presented in Table 1 and Fig. 1.

**Table 1:** Annual spray status of districts around the Lake Victoria basin from 2015 to 2017, showing number of structures sprayed and percentage of total structures sprayed

Region	District	2015	2016	2017
Kagera	Ngara	Sprayed 37,240 (98.7%)	Sprayed 52,885 (97.6%)	Sprayed 61,422 (97.3%)
	Biharamulo^a^	Sprayed 42,767 (93.3%)	Not sprayed	Not sprayed
	Muleba	Sprayed 81,294 (98.6%)	Not sprayed	Not sprayed
	Chato	Sprayed 53,899 (92.5%)	Sprayed 73,249 (95.8%)	Sprayed 83,163 (90.7%)
	Missenyi	Not sprayed	Sprayed 44,111 (97.3%	Sprayed 49,494 (97.3%)
	Bukoba Rural	Not sprayed	Sprayed 63,346 (99.4%)	Sprayed 69,083 (98.5%)
Mwanza	Magu	Sprayed 58,234 (91.8%)	Not sprayed	Not sprayed
	Misungwi	Sprayed 47,638 (92.4%)	Not sprayed	Not sprayed
	Sengerema	Not sprayed	Sprayed 97,012 (92.3%)	Sprayed 122,476 (94.6%)
	Kwimba	Not sprayed	Sprayed 71,733 (90.3%	Sprayed 90,634 (95.9%)
Simiyu	Busega^b^	Not sprayed	Not sprayed	Not sprayed
Mara	Rorya	Sprayed 77,228 (91.6%)	Not sprayed	Not sprayed
	Musoma Rural	Not sprayed	Sprayed 35,151 (95.8%)	Sprayed 40,981 (93.4%)
	Butiama	Not sprayed	Sprayed 50,066 (94.9%)	Sprayed 58,386 (94.3%)
	Tarime^a^	Not sprayed	Not sprayed	Not sprayed
Geita	Geita Town Council	Sprayed 21,363 (96.6%)	Sprayed (approx. 20,000 by Geita Gold Mine)	Sprayed (approx. 20,000 by Geita Gold Mine)
	Nyang'hwale	Not sprayed	Not sprayed	Sprayed 50,099 (95.5%)
	Bukombe^b^	Not sprayed	Not sprayed	Not sprayed
Mainland Total		419,753(94.2%)	487,553(94.9%)	625,738(95.1%)

^a^Control for 2017 entomological survey

^b^Control for 2016, 2017 entomological survey

### Residual efficacy of Actellic® 300CS (pirimiphos-methyl)

Cone bioassays were conducted on interior wall surfaces according to WHO protocols to determine the quality of spray within 14 days of application and the duration of residual efficacy, which was monitored monthly until mortality was lower than 80% for two consecutive months [14]. Batches of 2 to 5 days-old, non-blood-fed, female *An. gambiae s.s.* (Kisumu strain) were tested by exposing them to sprayed surfaces under WHO plastic cones for 30 min, after which they were transferred to clean paper cups and kept in a field insectary for recording delayed mortality. *Anopheles gambiae* Kisumu strain were known to be fully susceptible to pirimiphos-methyl and were reared in the National Institute for Medical Research Mwanza insectary at 27 ± 1 °C, and 60–80% relative humidity before being transported to the field in cool boxes for the assays. Knockdown and mortality were recorded 60 min post-exposure and after 24 h holding time. Portable untreated surfaces (approximately 30 cm by 30 cm) were constructed of cement, mud, burnt brick, whitewash, and painted substrates and used as negative controls. Cone tests on untreated portable surfaces were conducted outdoors (to avoid the airborne effect of Actellic® 300CS indoors) in a shaded area in parallel for each sprayed house. A summary of cone bioassay tests conducted is shown in Table 2.

**Table 2:** Overview of monthly cone bioassay in sprayed houses to determine residual efficacy

Year	No. of districts	No. of wall types tested ^(a)^	No. of houses tested p/month ^(b)^	Cone replicates p/month ^(c)^	No. of *An. gambiae* Kisumu tested p/month
2015	7	5	70	210	2100
2016	8	5	80	160	1600
2017	10	5	100	200	2000

(a) Five surface types of wall tested were mud, cement, painted, white wash and burnt brick. (b) There were at least 2 houses per surface type. (c) In 2015, 3 cones were placed on treated wall surfaces (1.5 m 1 m, 0.5 m); while in 2016 and 2017 only 2 cones were placed at 2 m and 1 m height from the floor, respectively

### Mosquito sampling and rainfall data

Three entomological sampling methods, indoor CDC light traps [15], indoor and outdoor CDC light trap fitted with bottle rotator (CBR) [16] and indoor Prokopack aspirators [17] were used in field mosquito collection. The number of sites, houses used for trapping, duration of sampling and outcomes are presented in Table 3. Rainfall data during the period of monthly observation on insecticide decay rate, vector densities, species composition, and *P. falciparum* infectivity were accessed from an online database system [18].

**Table 3:** Mosquito sampling methods, number of sites sampled, frequency of trapping and outcomes

Method	Sites	Number of houses/ traps	Frequency and year	Outcomes
CDC light trap	10 IRS sites + 4 control sites	2 houses per site per night; 1 light trap per house per night	28 nights per month 2016–17	Species composition and indoor vector abundance
CDC Light trap fitted with bottle rotator (CBR)	4 IRS sites + 4 control sites	10 houses per site per month; 2 CBRs per house per night (one indoors and one outdoors)	10 nights per month 2017	Species composition Biting pattern / activity Blood meal analysis
Prokopack aspirator	4 IRS sites + 4 controls	10 houses per site per month	20 days per month 2017	Species composition Indoor resting density

### Indoor CDC light traps

In 2016 and 2017, two houses per night were selected for CDC light traps on 28 consecutive nights each month (for a total of 56 trap nights per month per site). The same houses were used for sampling per site every month. House selection was based on a random pick of houses near the residence of community mosquito collectors. In selected houses, CDC light traps were installed indoors, *circa* 1.5 m above the floor next to the head of the sleeping person [19]. The person(s) was requested to sleep under an intact untreated mosquito net(s) provided by the project. CDC light traps were set to operate from 18:00 to 06:00. In the morning, captured mosquitoes were transferred into labelled paper cups and taken for preliminary morphological identification in the field office. All mosquitoes from traps were killed before conducting morphological identification and recording results according to species, sex and abdominal status.

### CDC light trap with collection bottle rotator (CBR)

One CDC light trap with automatic collection bottle rotator (CBR—John Hock model 1512) was set indoors and one outdoors at 10 randomly selected houses each site for 10 nights per month. CBR traps were set from March to December (10 months) in 2017 and sampling was scheduled on nights near a new moon to minimize the effect of moonlight on the outdoor light-trap collection, and to reduce bias when comparing species distribution across seasons. An estimate of the presence and period of moonlight was calculated using an online lunar calendar [20]. Indoor CBRs were set up in sleeping areas of houses, while outdoor CBRs were set up within a 10-m radius of the house. Ethical concerns restrict use of human landing collection (HLC) for mosquito collection. Therefore, the CBR trapping was considered a proxy for HLC, targeting host-seeking malaria vector mosquitoes. Indoor and outdoor human-biting rate of *Anopheles* and time of biting were determined in the selected sentinel sites. All mosquitoes from traps were killed before conducting morphological identification and recording results according to species, sex and abdominal status.

### Indoor Prokopack aspirator

The improved Prokopack aspirator (John Hock model 1419) was used for sampling indoor resting mosquitoes from 10 houses daily over 20 days within each selected sentinel site per month in 2017 [17, 21]*.* Aspiration was carried out in the morning between 06:00 and 08:00 and was conducted in all rooms (range of 2 to 4 rooms per house, with each room having up to 3 occupants) in the house, moving the aspirator across walls, ceiling and near furniture. To standardize the collection, the sampling was conducted for a total of 30 min per house, by two assistants working simultaneously in the same house for 15 min each. Long white door curtains were used to cover the door space during aspiration to prevent mosquitoes from exiting.

### Laboratory analysis

Collected samples were identified to species morphologically using the systematic key of Gillies and Coetzee [22]. A sub-sample of 8,957 female anopheline mosquitoes collected in 2016 and 2017 were subsequently analysed for presence of *P. falciparum* sporozoites by enzyme-linked immunosorbent assay (ELISA) technique according to the protocol of Burkot et al. [23] and slightly modified by Wirtz et al. [24]. Polymerase chain reaction (PCR) was conducted to identify members of the *An. gambiae* complex and *Anopheles funestus* group according to the protocols of Scott et al. and Wilkins et al*.* [25–27]. Blood-fed *Anopheles* collected from Prokopack aspirators, CDC light traps and CBRs were tested for blood meal source, using the ELISA protocol described by Beier et al. [28].

### Data analysis

The mortality rates from monthly cone bioassay monitoring of Actellic® 300CS (pirimiphos-methyl) were corrected using Abbot’s formula when mortality in negative controls was between 5 and 20%. Whenever untreated control mortality was above 20% the results were discarded and the tests repeated [29]. Indoor vector resting density was calculated as the total number of female *Anopheles* collected (by species), divided by the total number of rooms surveyed by Prokopack aspirator. The human biting rate was calculated as the total number of mosquitoes collected by CDC light trap, divided by the number of trap nights. Sporozoite rate was estimated as the proportion of female *Anopheles* found positive for the presence of circumsporozoite proteins. Sporozoite rates in unsprayed and sprayed sites were compared by Kruskal–Wallis Chi square test to determine the infectivity rates of *An. gambiae* and *An. funestus s.s.* All statistical tests with 95% confidence intervals were calculated using RStudio; with R version 3.4.4.

## Results

### Residual efficacy of Actellic® 300CS (pirimiphos-methyl), 2015–17

**Fig. 2:**
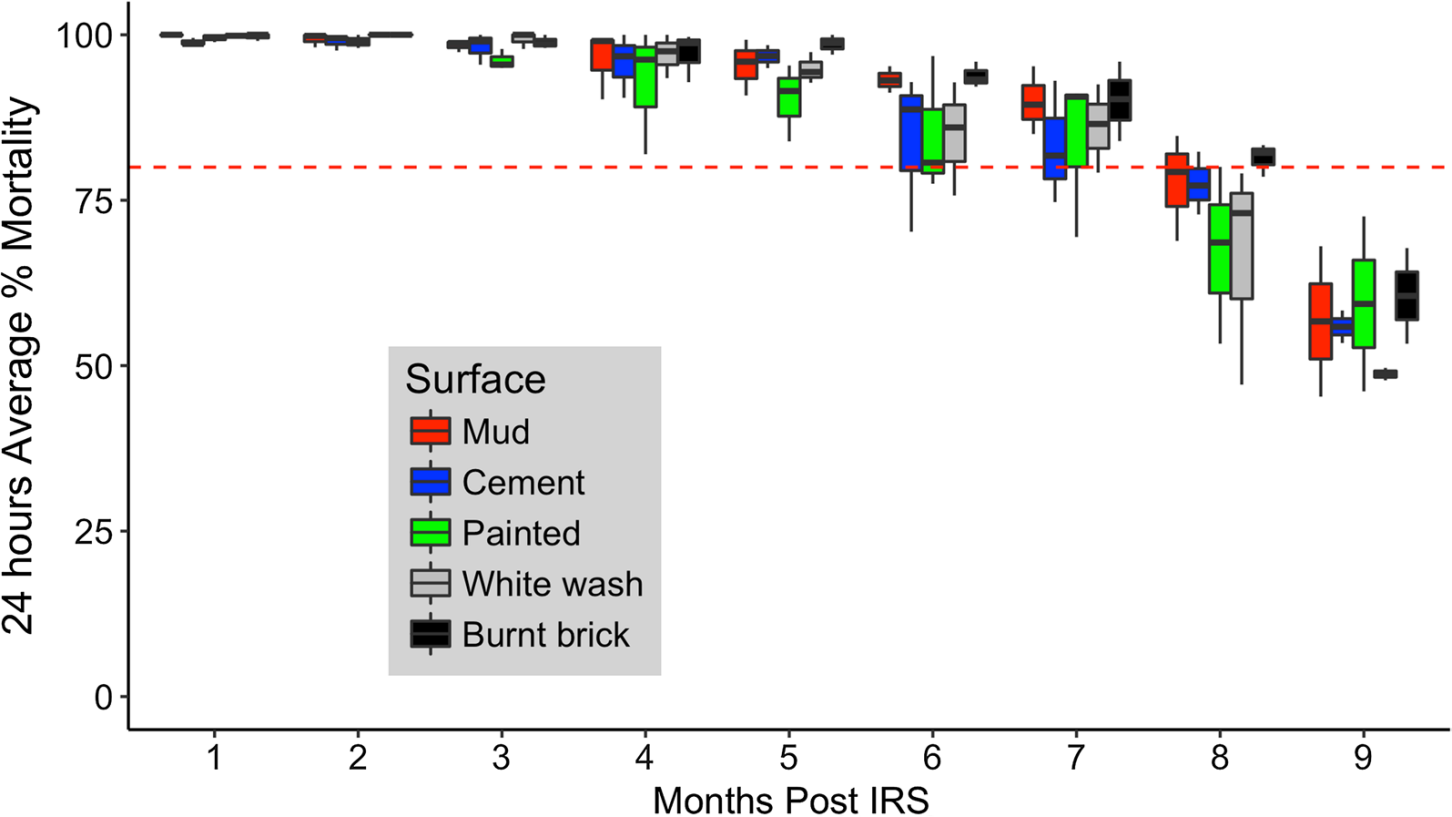
Mean monthly percentage mortality (24 h) of *Anopheles gambiae* (Kisumu). Mean represents 24 h mortality after 30 min cone bioassay on mud, cement, painted, white wash, and burnt brick walls that were sprayed with Actellic® 300CS in 2015, 2016 and 2017. The red dotted line shows the WHO standard cut-off (80% mortality)

Overall results shortly after spraying showed that the quality of spraying in 2015, 2016 and 2017 was satisfactory and mortality rates were consistent across all wall surface types sprayed by different spray operators and teams. All tests conducted < 2 weeks after spray application resulted in mortality of 100%, with the exception of a lowest mortality recorded at 90.8% from one house in 2016. Monthly cone bioassay indicated a mean residual duration of 7 months post-spraying (mortality > 80% WHO defined mortality threshold), with a decrease in mortality to approximately 50–70% recorded 9 months post-spraying (Fig. 2). Trends were similar for all wall substrates.

### Vector seasonality

#### Indoor density of *Anopheles gambiae * sensu lato (*s.l. *) and *Anopheles funestus s.l. * by CDC light trap (2016–17)

**Fig. 3:**
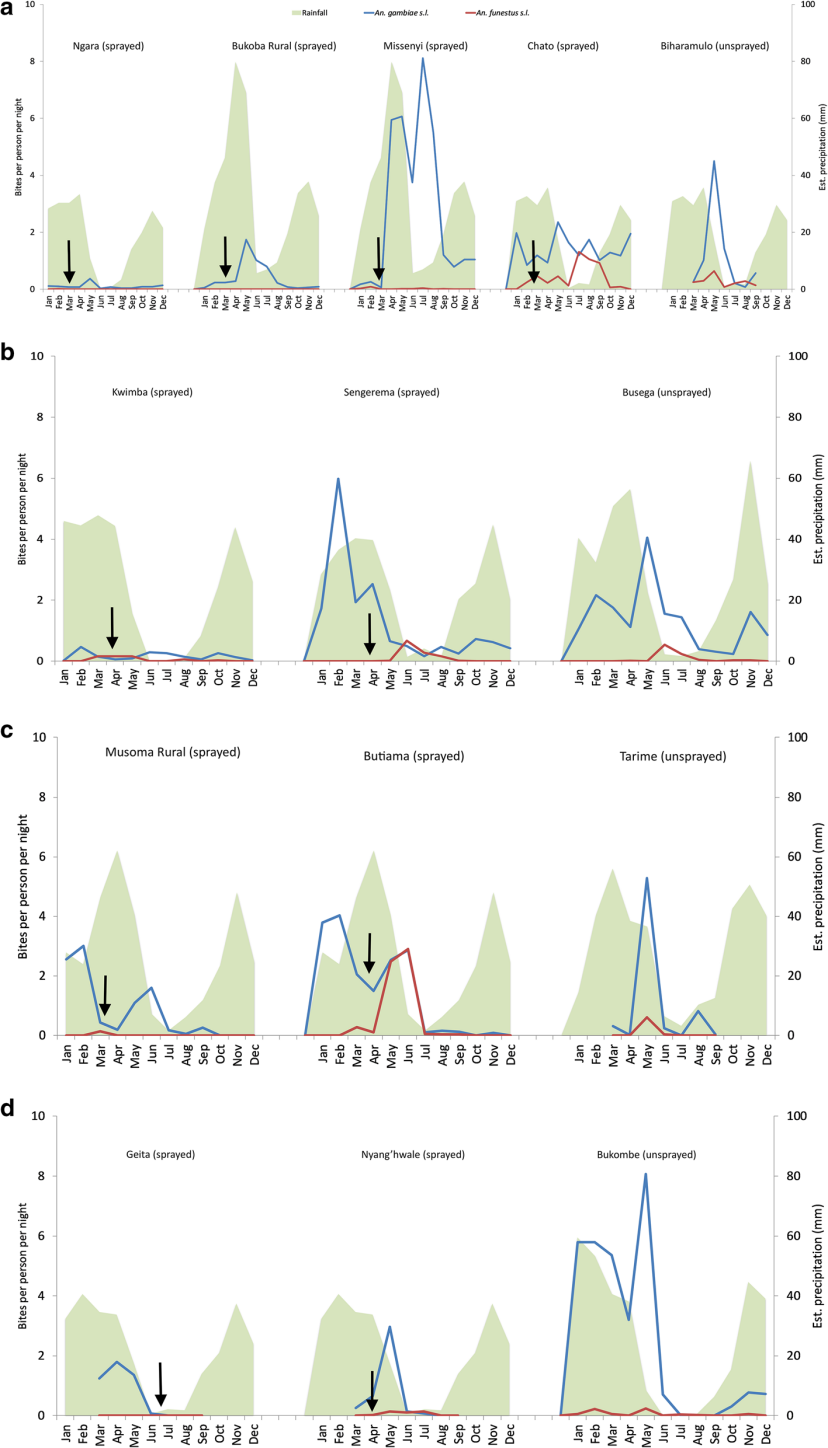
Mean nightly indoor catch of *Anopheles gambiae s.l.* and *Anopheles funestus s.l.* Indoor density of *An. gambiae s.l.* and *An. funestus s.l.* collected from CDC light traps for sampling period 2016–2017 (except for Nyang’hwale (sprayed, 2017) and Tarime (unsprayed, 2017) where data were collected in 2017 only). **a** Kagera region with Biharamulo as control site. **b** Mwanza region with Busega (a close by site, in Simiyu region, as a control site. **c** Mara region with Tarime as control site. **d** Geita region with Bukombe as control sites. Arrows indicate time when IRS was conducted

Figure 3 presents the mean nightly indoor catch of *An. gambiae s.l.* and *An. funestus s.l.* from indoor CDC light trap collections conducted monthly for 2 years from January 2016 to December 2017. *Anopheles gambiae s.l.* was the predominant vector species in all sites throughout the sampling period over the 2-year period 2016–2017. *Anopheles funestus s.l.* indoor densities were very low in most sites, with relatively high indoor densities only recorded in Chato (June-October) and Butiama (May–June). Density peaks were generally observed following periods of significant rainfall occurring between October and April (Fig. 3).

Following IRS in February/March, indoor densities were generally low in sprayed sites, at < 3 *An. gambiae s.l.* per trap/night between March and December (1–10 months after spraying). In the sprayed sites of Ngara (Kagera Region), Geita (Geita Region) and Kwimba (Mwanza Region), densities were particularly low year-round, never exceeding 1 per trap/night. However, in Missenyi (Kagera Region) indoor *An. gambiae s.l.* densities were particularly high between April and August at 4–8 per trap/night, despite IRS in February. While in Butiama a smaller indoor peak of *An. funestus s.l.* was reached in June at 3 per trap/night, 3 months after IRS.

In many sprayed sentinel sites, including Chato (Kagera Region), Sengerema (Mwanza Region), Musoma Rural and Butiama (Mara Region) relatively high *An. gambiae s.l.* indoor densities were recorded between January and February. This is 10–12 months after the previous IRS cycle, by which point residual efficacy had waned.

#### Biting rate for *An. gambiae s.l.* using CDC light trap fitted with bottle rotator (CBR)

In 2017, CBR traps were set from March to December. Data were combined for 4 sprayed sites (Sengerema, Musoma Rural, Chato, Bukoba Rural), and 4 unsprayed sites (Busega, Bukombe, Tarime, Biharamulo) to compare the mean biting rate indoors and outdoors. The total catch size per site using CBR (indoors and outdoors) over 200 trap nights per site indoors and outdoors (10 trap nights per month both indoors and outdoors for 10 months) was 4616 *An. gambiae s.l.* from sprayed sites and 5260 from unsprayed sites. The total *An. gambiae s.l.* collected per sprayed site was 333 from Sengerema, 290 from Musoma Rural, 3809 from Chato, and 184 from Bukoba Rural; while for unsprayed sites the total was 83 from Tarime, 2795 from Bukombe, 2303 from Biharamulo, and 79 from Busega.

**Fig. 4:**
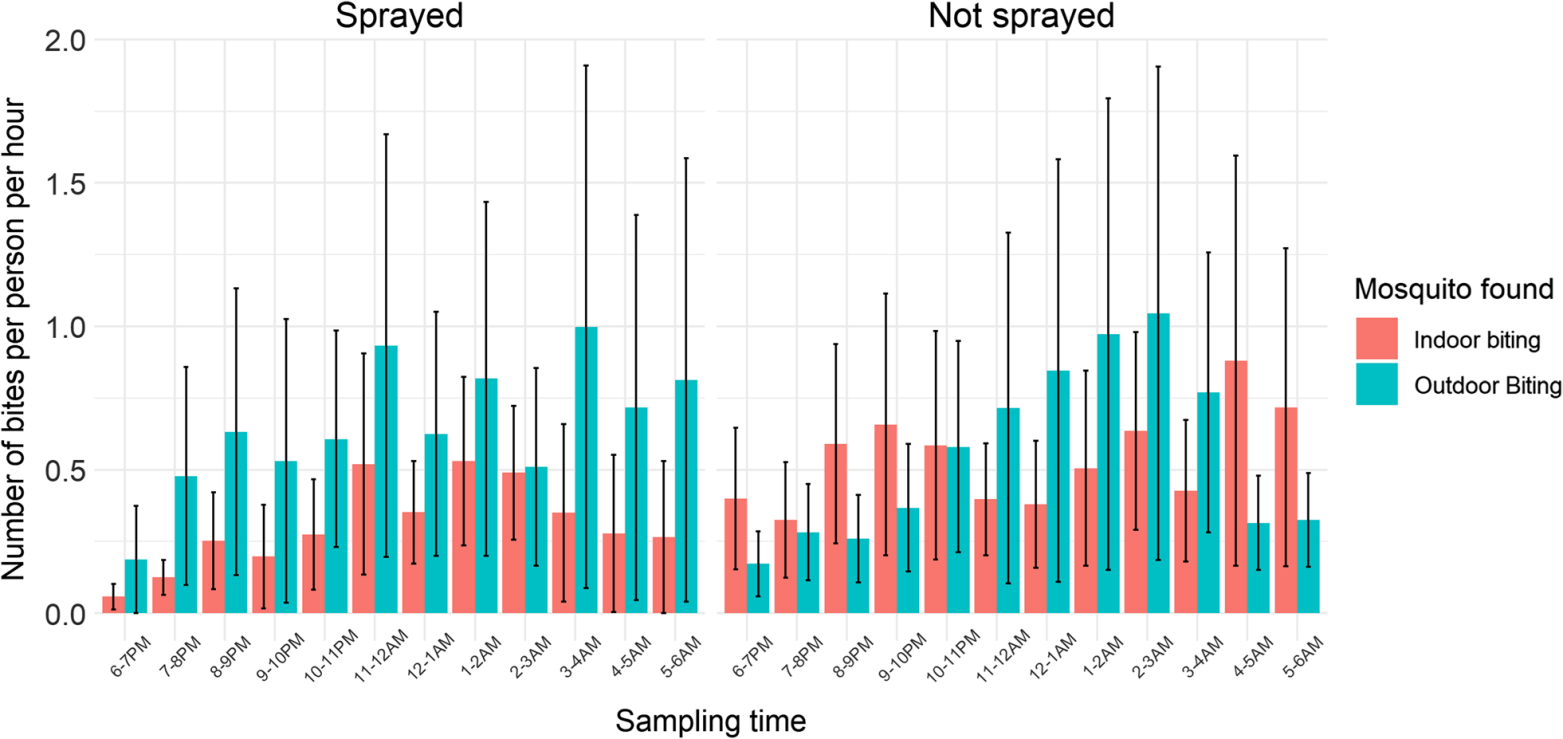
Biting rate for *Anopheles gambiae s.l.* Mean biting time of *An. gambiae s.l.* from CBR conducted indoors and outdoors. Mean hourly biting rate is indicated from 18:00 to 06:00 in sprayed and unsprayed areas

In sprayed sites, the *An. gambiae s.l.* biting rate was higher outdoors than indoors at all times of night. In unsprayed sites there was more outdoor biting, but only late at night between 22.00 and 03.00. However, it should be noted that the majority of *An. gambiae s.l.* in all sites were collected later in the evening when the majority of people are likely to be indoors and protected by LLINs. Nevertheless, a greater degree of outdoor biting risk was observed early in the evening in sprayed sites compared to unsprayed sites (Fig. 4).

#### Indoor resting densities of *An. gambiae s.l.* in 2017 using Prokopack aspirators

**Fig. 5:**
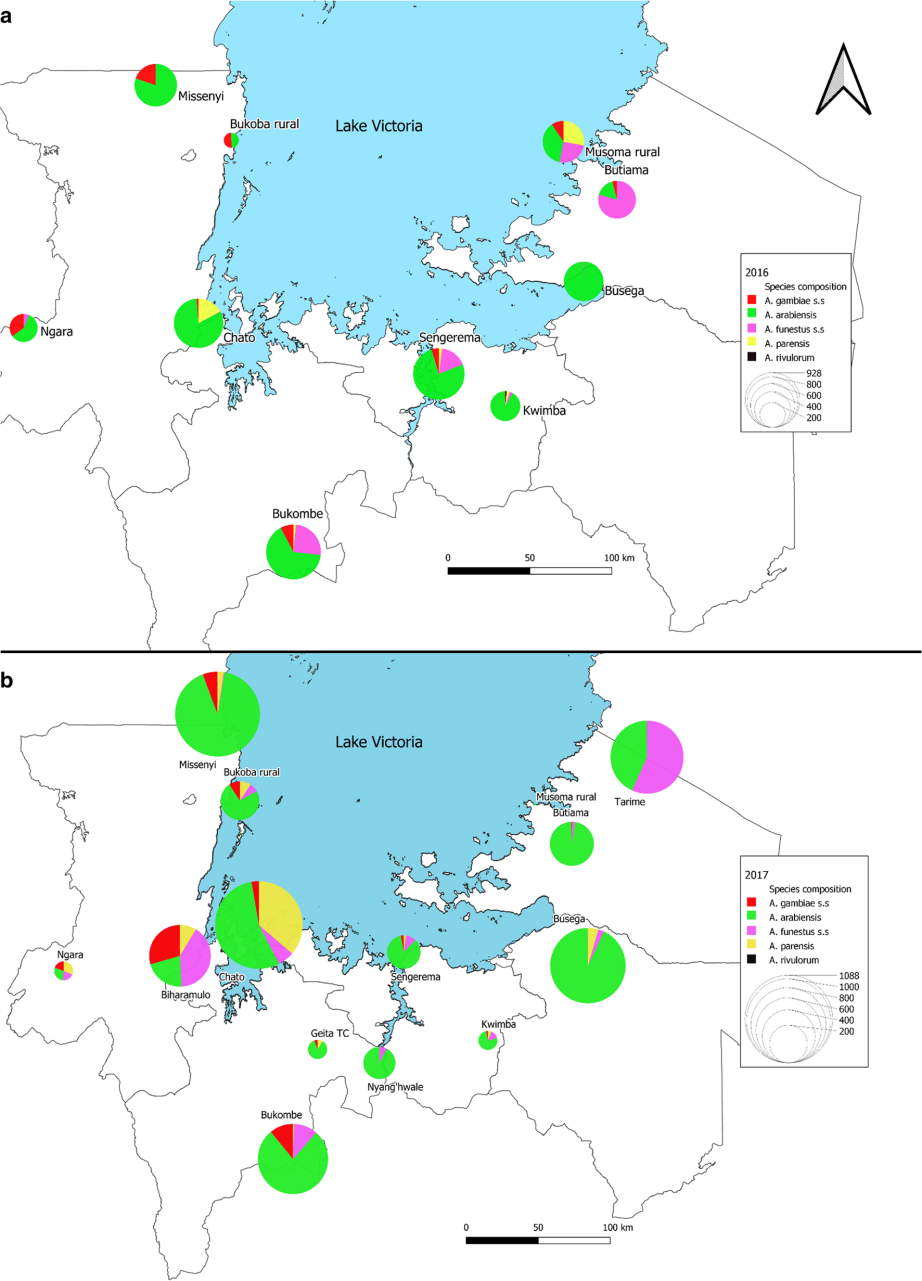
Mosquito species composition. Species composition expressed as a proportion of *Anopheles* species tested by PCR in respective years **a** 2016 **b** 2017. In 2016, 8 of 10 sites were sprayed with Actellic® 300CS; in 2017, 9 of 13 sites were sprayed with Actellic® 300CS

**Fig. 6:**
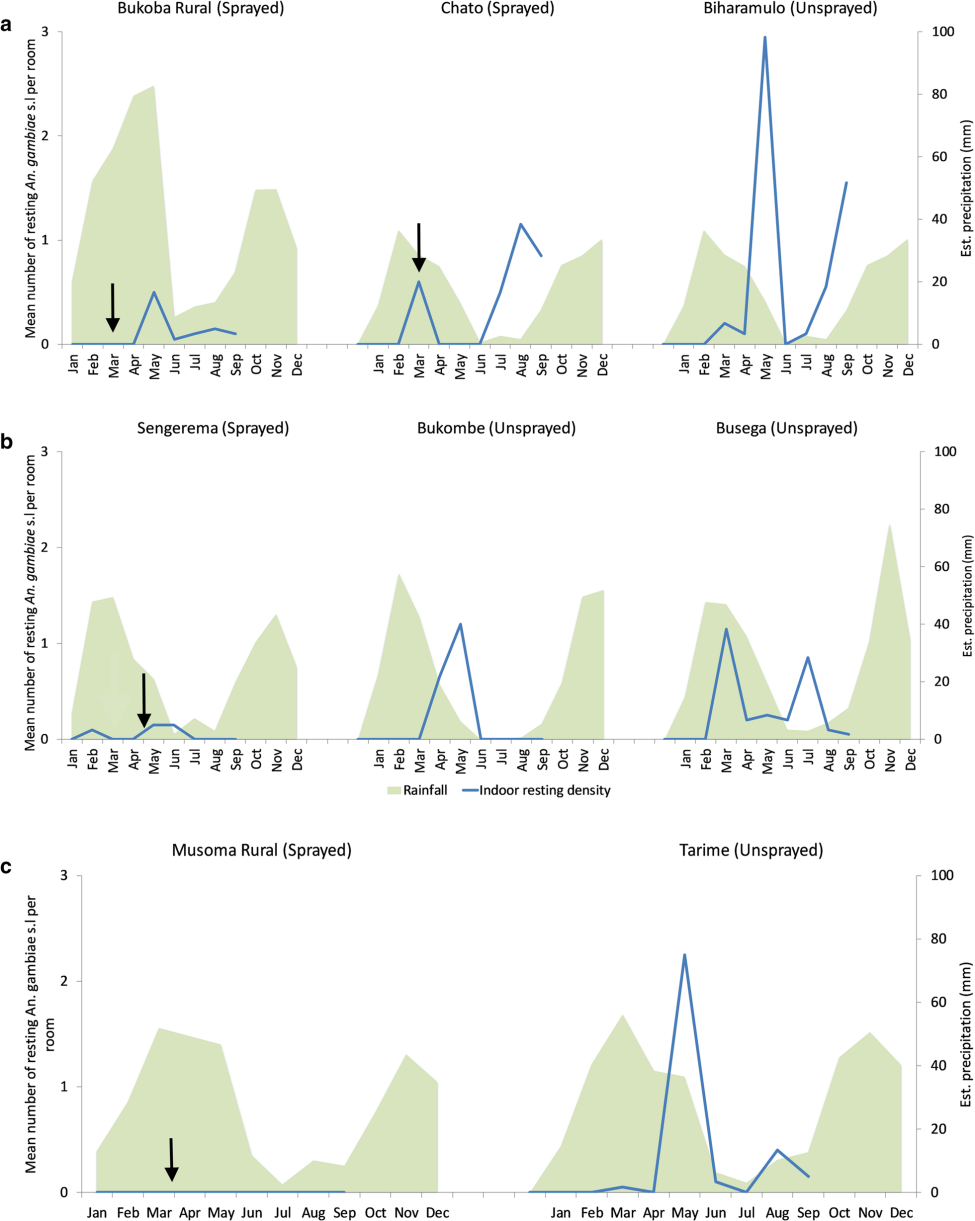
Indoor resting density for *Anopheles gambiae s.l.* **a** Kagera region with Biharamulo as control. **b** Geita, and Mwanza region with Bukombe and Busega as controls, respectively. **c** Mara region with Tarime as control. Arrows indicate time when IRS was conducted. Analysis of variance indicate significantly higher (p < 0.05) indoor resting densities across regions (i.e., Kagera, Geita, Mwanza, Mara)

The mean number of *An. gambiae s.l.* collected by Prokopack aspirator resting indoors was greater in the 4 unsprayed sites of Biharamulo, Bukombe, Busega, and Tarime than in the 4 sprayed sites of Bukoba Rural, Chato, Sengerema, and Musoma Rural. In general, the highest peak in resting density was observed between May and August after the long rain season, with Chato and Busega also having a smaller peak in March (Fig. 5). There was no *An. gambiae s.l.* collected throughout the 2017 collection period in the sprayed site of Musoma Rural (Fig. 6).

### Species composition

A total of 8,957 female *Anopheles* collected from 2016 to 2017 were analysed by PCR for species identification. The samples consisted of 5,306 (59.2%) from sprayed and 3,651 (40.8%) from unsprayed sites, with 4,389 (49%), 2,866 (32%), and 1,702 (19%) collected from CDC light trap, CBR and Prokopack aspirator, respectively. Results confirmed vector populations in sprayed districts to be predominantly *Anopheles arabiensis* (71%, n = 3,768) with minor proportions of *Anopheles parensis* (11,1%, n = 589), *An. funestus s.s.* (11%, n = 585, *An. gambiae s.s.* (6.8%, n = 361), and *Anopheles rivulorum* (0.1%, n = 3). There was a significantly greater mean number per year of *An. funestus s.s.* in unsprayed sites than sprayed sites (214.5 *vs* 58.5, p = 0.024). The predominant vector species in unsprayed districts was *An. arabiensis* (66.9%, n = 2441), however there was a higher proportion of *An. funestus s.s.* (23.5%, n = 858) and fewer *An. parensis* (2.3%, n = 85), and similar proportion of *An. gambiae s.s*. (7.3%, n = 267) as in sprayed sites.

### Sporozoite rate

Between 2016 and 2017 the overall *P. falciparum* sporozoite rate across all sites (sprayed and unsprayed) for all *Anopheles* (*An. funestus*, *An. arabiensis*, *An. gambiae*, and *An. parensis*) combined was estimated as 1.72% (286/16,670). The overall sporozoite infection rate was higher in unsprayed sites, estimated as 2.02% (115/5,686) than in sprayed sites at 1.56% (171/10,984) (Kruskal–Wallis test, *H* (3) = 6.584, *p* = 0.086). Mean sporozoite rates were generally less than 2% for all sprayed sites (from 2016 to 2017), with the highest rates scored at 4.5% (Ngara, 2017) and 3.9% (Biharamulo, 2016) in areas where *An. funestus* and *An. gambiae* were relatively common. See Additional file 1: Table S1 for 2016 and 2017 sporozoite rates presented by site.

Results from 2017 were disaggregated by species (from PCR results) and spray status (Table 4). This could not be done with data from 2016. Results by species showed that *An. funestus s.s.* had the highest sporozoite rate estimated at 4.07% (30/738) across unsprayed and sprayed sites combined. The mean *An. funestus s.s.* sporozoite rate estimated as 4.3% (27/630) in unsprayed sites and 2.8% (3/108) in sprayed sites, although the difference was not statistically significant (*p* = 0.48) (Table 4), possibly due to the small sample size in sprayed sites. The predominant species, *An. arabiensis* exhibited a relatively lower overall sporozoite rate in 2017 estimated as 1.34% (45/3,366), with a higher sporozoite rate in unsprayed sites (2.0%; with 95% CI 1.4–2.9) compared to sprayed sites (0.8% with 95% CI 0.5–1.3) (p = 0.003). Although not commonly considered as an important malaria vector, *An. parensis* had an overall sporozoite rate of 1.1% (5/435).

**Table 4:** Sporozoite rates disaggregated by vector species and spray status from 2017 sampling

Mosquito species	Spray status	No. of samples analyzed	Number sporozoite positive	Sporozoite rate % (95% CI)	P-value
*An. gambiae* s.s	Sprayed	118	3	2.5 (0.5–7.2)	0.876
	Unsprayed	177	5	2.8 (0.9–6.5)	
*An. arabiensis*	Sprayed	1924	16	0.8 (0.5–1.3)	0.003
	Unsprayed	1442	29	2.0 (1.3–2.9)	
*An. funestus* s.s	Sprayed	108	3	2.8 (0.6–7.9)	0.480
	Unsprayed	630	27	4.3 (2.8–6.2)	
*An. parensis*	Sprayed	362	4	1.1 (0.3–2.8)	0.840
	Unsprayed	73	1	1.4 (0.03–7.4)	

### Blood meal analysis

A total of 194 blood-fed *An. arabiensis* (identified by PCR) that were collected from January to September 2017 by indoor resting collections were tested for vertebrate host blood source (human, bovine, goat, dog) with 109 from sprayed sites (Sengerema, Kwimba, Bukoba rural, Missenyi) and 85 from unsprayed sites (Bukombe and Busega). Overall, the proportion of *An. arabiensis* that fed on humans (including mixed blood meals on both human and animal) was 59.3% (115/194), with cattle blood being the most common non-human source. The overall human blood index was 0.62 in sprayed sites and 0.55 in unsprayed sites. An estimated 32.5% (63/194) of *An. arabiensis* fed on both human and animals, demonstrating opportunistic feeding behaviour, while only 26.8% fed only on humans (Additional file 1: Table S2).

## Discussion

Cone bioassay results following IRS with Actellic® 300CS show that the residual efficacy in northwestern Tanzania was a mean of 7 months. Seven months residual duration lies in the higher end of performance for this insecticide formulation, considering a range of 2–9 months that was observed in 9 other PMI-supported countries [30]. Although *An. arabiensis* in the Lake zone of northwestern Tanzania are resistant to pyrethroids, with high intensity resistance present in some sites, they were susceptible to pirimiphos-methyl during the study [7].

IRS campaigns were usually conducted in February and March, meaning that protection was provided through the year up to October/November. However, rainfall in northwestern Tanzania is bi-modal, with a second peak of *An. gambiae s.l.* occurring in January and February, which is 10–12 months after the previous IRS cycle, by which time insecticide efficacy had decreased substantially. In response to entomology data from this study and District Health Information System 2 (DHIS2)-derived reports on peak malaria cases, the timing of IRS has since been changed to November in Kagera and Geita Regions in 2018 [31]. Spraying towards the end of the year should provide better protection during the two major malaria peaks of December/January and June/July.

Consistent with results from other studies in neighbouring western Kenya [32], the peak biting rates of *An. gambiae s.l.* were observed to occur in unsprayed sites late at night, although was higher outdoors than indoors. Biting time and location (indoors/outdoors) can change depending on host availability [33] and selection for outdoor biting due to indoor insecticide exposure. Reported results from this study suggest that *An. gambiae s.l.* (mostly *An. arabiensis*) may have shifted to bite more often outdoors in sites where IRS has been conducted for several years [34]. *Anopheles arabiensis* were shown to be opportunistic in feeding behaviour, with many having fed on both human and animal hosts (mostly cattle). This may partially explain why *An. arabiensis* was the predominant malaria vector species collected in sprayed sites, with *An. gambiae s.s.* and *An. funestus s.s.* more readily controlled due to their anthropophilic and endophilic nature [32, 35]. However, *An. arabiensis* was also the predominant species in unsprayed sites, indicating that other factors including climatic conditions and other control measures (particularly LLINs) have contributed to *An. arabiensis* dominating in this region.

*Anopheles funestus* had the highest sporozoite rate among all species of *Anopheles* collected in 2017, but only constituted 8% of *Anopheles* collected in sprayed sites and 20% in unsprayed sites. Reported results from this study suggest that there was a higher sporozoite carriage by *An. funestus* in unsprayed sites in comparison to sprayed sites, while there was also evidence for a species shift in sprayed sites within the *An. funestus* group. In some sprayed sites in Kagera *An. parensis* (member of the *An. funestus* group) replaced *An. funestus s.s*., as was reported in coastal Kenya, following IRS with DDT in the 1960s [36]. IRS has been extremely successful in controlling *An. funestus s.s.* in several countries, with the species being highly anthropophilic and preferring to rest indoors. In the Pare/Taveta area of East Africa, where dieldrin was sprayed between 1954–1959 *An. funestus* complex was not found for 3 years after the end of spraying but the more zoophilic species *An. rivulorum* (of the *An. funestus* group) became common thereafter [37]. The finding of *An. parensis* with sporozoites indicates that this species is probably becoming an important secondary vector [38] in Tanzania, as has been demonstrated in South Africa [39].

One of the limitations of this study is that there was no baseline monitoring of vector densities and sporozoite rates before IRS was first conducted in each site. There were also few unsprayed sites, which were relatively far from sprayed sites. These two factors make it difficult to directly determine the impact that IRS had on vector populations. Nevertheless, in the majority of sites, IRS with pirimiphos-methyl CS was successful in keeping vector densities relatively low for approximately 9 months after spraying. There was an exception in the sprayed site of Missenyi, where a particularly high density of *An. gambiae s.l.* was collected just a few months after spraying. Missenyi district is known to receive a relatively high amount of rainfall in March–May and most arable land is used for sugar cane cultivation that results in prolonged availability of larval habitats for anophelines.

## Conclusion

IRS had a substantial impact on malaria transmission, with the sporozoite rate in the predominant malaria vector species, *An. arabiensis*, being 59% lower in sprayed sites than in unsprayed sites in 2017. This is in keeping with a study in Kagera Region which showed that a combination of non-pyrethroid IRS together with pyrethroid LLINs resulted in fewer cases of malaria than villages with LLINs only [40].

## Supplementary information

**Additional file 1: Table S1.** Sporozoite rate and entomological inoculation rate (all *Anopheles* tested) in all 14 districts for 2016 and 2017.** Table S2.** Results of ELISA to determine blood meal source of *Anopheles* *arabiensis* collected by Prokopack aspirator and CDC light trap.

## Data Availability

The datasets used and/or analysed during the current study are available from the corresponding author on reasonable request.

## Abbreviations

CBR: Collection bottle rotators
CDC: Centers for Disease Control
EIR: Entomological inoculation rate
HBR: Human biting rate
ICON 10 CS: Lambda-cyhalothrin capsule suspension
ITNs: Insecticide-treated nets
IRS: Indoor residual spraying; DDT: 4,4′-(2,2,2trichloroethane-1,1-diyl) bis (chlorobenzene)
KDT: Knock down time
LLIN: Long-lasting insecticidal net
MoHCDGEC: Ministry of Health, Community Development, Gender, Elderly and Children
PMI: President’s Malaria Initiative
WHO: World Health Organization
