# Supplementary material for: Malaria vector species composition and entomological indices following indoor residual spraying in regions bordering Lake Victoria, Tanzania
Source: Malar J. 2020 Oct 28;19:383. doi: 10.1186/s12936-020-03452-w (PMC7594290; doi:10.1186/s12936-020-03452-w)
Supplement: Supplementary file 1 — Additional file 1: Table S1. Sporozoite rate and entomological inoculation rate (all Anopheles tested) in all 14 districts for 2016 and 2017. Table S2. Results of ELISA to determine blood meal source of Anopheles arabiensis collected by Prokopack aspirator and CDC light trap. [file 12936_2020_3452_MOESM1_ESM.docx]

**Additional file 1: Table S1** Sporozoite **rate and entomological inoculation rate (all *Anopheles* tested) in all 14 districts for 2016 and 2017**

*denotes unsprayed control sites.

| Sporozoite rate (SR) (%) = (total positive/total tested)  Human biting rate (HBR) = (total collected in CDC-LT/total trap nights)  Nightly entomological inoculation rate (EIR) = (Human biting rate (HBR) x SR | | | | | | | | | |
| --- | --- | --- | --- | --- | --- | --- | --- | --- | --- |
| Region | **District** | **SR 2016** | **Mean HBR per night 2016** | **Mean nightly EIR 2016** | **Annual EIR 2016** | **SR 2017** | **Mean HBR per night 2017** | **Mean nightly EIR 2017** | **Annual EIR 2017** |
| Kagera | Ngara | 4.5% (10/220) | 0.09 | 0.004 | 1.49 | 0.0% (0/50) | 0.128 | 0.000 | 0.00 |
|  | Chato | 1.2% (22/1,810) | 1.25 | 0.015 | 5.55 | 1.1% (12/1,091) | 1.701 | 0.019 | 6.83 |
|  | Missenyi | 1.6% (22/1,357) | 3.20 | 0.051 | 18.94 | 0.8% (9/1,066) | 2.961 | 0.024 | 9.12 |
|  | Bukoba rural | 1.3% (4/310) | 0.41 | 0.005 | 1.93 | 1.1% (3/279) | 0.512 | 0.006 | 2.01 |
|  | Biharamulo* | N/A | N/A | N/A | N/A | 3.9% (22/560) | 1.151 | 0.045 | 16.50 |
| Mwanza | Sengerema | 0.9% (13/1,422) | 1.95 | 0.018 | 6.51 | 1.9% (3/158) | 0.752 | 0.014 | 5.21 |
|  | Kwimba | 2.3% (9/386) | 0.19 | 0.004 | 1.62 | 0.0% (0/52) | 0.137 | 0.000 | 0.00 |
| Simiyu | Busega* | 1.4% (8/586) | 0.89 | 0.013 | 4.43 | 1.1% (9/806) | 2.188 | 0.024 | 8.92 |
| Mara | Musoma rural | 2.2% (31/1,416) | 1.52 | 0.033 | 12.15 | 0.0% (0/5) | 0.06 | 0.000 | 0.00 |
|  | Butiama | 2.9% (26/888) | 1.73 | 0.050 | 18.49 | 1.8% (5/276) | 1.512 | 0.027 | 10.00 |
|  | Tarime* | N/A | N/A | N/A | N/A | 2.2% (17/772) | 0.962 | 0.021 | 7.73 |
| Geita | Geita TC | N/A | N/A | N/A | N/A | 1.9% (1/52) | 0.638 | 0.012 | 4.48 |
|  | Nyang’hwale | N/A | N/A | N/A | N/A | 1.4% (2/146) | 0.583 | 0.008 | 2.92 |
|  | Bukombe* | 1.7% (38/2,250) | 3.73 | 0.063 | 22.99 | 2.9% (21/712) | 1.662 | 0.048 | 17.89 |

**Additional file 2: Table S2** **Results of ELISA to determine blood meal source of *Anopheles* *arabiensis* collected by Prokopack aspirator and CDC light trap**

| **District** | **No. tested** | **Human** | **Cow** | **Goat or dog** | **Mixed**  **(human + animal)** | **Mixed (animal + animal)** | **Human blood index** |
| --- | --- | --- | --- | --- | --- | --- | --- |
| Sengerema | 13 | 3 | 1 | 0 | 3 | 6 | 0.46 |
| Missenyi | 78 | 21 | 18 | 0 | 30 | 9 | 0.65 |
| Bukoba Rural | 17 | 4 | 0 | 4 | 6 | 3 | 0.59 |
| Kwimba | 1 | 1 | 0 | 0 | 0 | 0 | 1.00 |
| Total sprayed sites | 109 | 26.6%  (29) | 17.4%  (19) | 3.7%  (4) | 35.8%  (39) | 16.5%  (18) | 0.62 |
| Bukombe | 37 | 6 | 11 | 1 | 2 | 17 | 0.22 |
| Busega | 48 | 17 | 4 | 0 | 22 | 5 | 0.81 |
| Total unsprayed sites | 85 | 27.1%  (23) | 17.6%  (15) | 1.2%  (1) | 28.2%  (24) | 25.9%  (22) | 0.55 |
